# Worldwide variations in COVID-19 vaccination policies and practices in liver transplant settings: results of a multi-society global survey

**DOI:** 10.3389/frtra.2023.1332616

**Published:** 2024-01-19

**Authors:** Tommaso Di Maira, Carmen Vinaixa, Manhal Izzy, Francesco Paolo Russo, Varvara A. Kirchner, Ashwin Rammohan, Luca Saverio Belli, Wojciech Grzegorz Polak, Thomas Berg, Marina Berenguer

**Affiliations:** ^1^ Liver Transplantation and Hepatology Unit, Hospital Universitari I Politècnic La Fe, Valencia, Spain; ^2^ Biomedical Research Network Center for Hepatic and Digestive Diseases (CIBEREHD), Madrid, Spain; ^3^ La Fe Health Research Institute, Valencia, Spain; ^4^ Department of Medicine, Division of Gastroenterology, Hepatology, and Nutrition, Vanderbilt University Medical Center, Nashville, TN, United States; ^5^ Department of Surgery, Oncology and Gastroenterology, University Hospital Padua, Padua, Italy; ^6^ Division of Abdominal Transplantation, Department of Surgery, Stanford University, Stanford, CA, United States; ^7^ The Institute of Liver Disease & Transplantation, Dr. Rela Institute & Medical Centre, Bharath Institute of Higher Education and Research, Chennai, India; ^8^ Department of Hepatology and Gastroenterology, Niguarda Hospital, Milan, Italy; ^9^ Department of Surgery, Division of HPB and Transplant Surgery, Erasmus MC Transplant Institute, University Medical Centre, Rotterdam, Netherlands; ^10^ Division of Hepatology, Department of Medicine II, Leipzig University, Medical Center, Leipzig, Germany

**Keywords:** vaccine, SARS-CoV-2, liver, transplant, survey, policy, side effect, worldwide activity

## Abstract

**Background:**

Despite the WHO's report of 24 available SARS-CoV-2 vaccines, limited data exist regarding vaccination policies for liver transplant (LT) patients. To address this, we conducted a global multi-society survey (EASL-ESOT-ELITA-ILTS) in LT centers.

**Methods:**

A digital questionnaire assessing vaccine policies, safety, efficacy, and center data was administered online to LT centers.

**Results:**

Out of 168 responding centers, 46.4%, 28%, 13.1%, 10.7%, and 1.8% were from European, American, Western Pacific, Southeast Asian, and Eastern Mediterranean Regions. Most LT centers prioritized COVID-19 vaccine access for LT patients (76%) and healthcare workers (86%), while other categories had lower priority (30%). One-third of responders recommended mRNA vaccine exclusively, while booster doses were widely recommended (81%). One-third conducted post-vaccine liver function tests post COVID-19 vaccine. Only 16% of centers modified immunosuppression, and mycophenolate discontinuation or modification was the main approach. Side effects were seen in 1 in 1,000 vaccinated patients, with thromboembolism, acute rejection, and allergic reaction being the most severe. mRNA showed fewer side effects (−3.1, *p* = 0.002).

**Conclusion:**

COVID-19 vaccines and booster doses were widely used among LT recipients and healthcare workers, without a specific vaccine preference. Preventative immunosuppression adjustment post-vaccination was uncommon. mRNA vaccines demonstrated a favorable safety profile in this population.

## Introduction

Severe acute respiratory syndrome coronavirus 2 (SARS-CoV-2) is an RNA virus that was first described in Wuhan, China, in December 2019 (1). Since then, SARS-CoV-2 has spread globally, causing the coronavirus disease 2019 (COVID-19) pandemic, resulting in >611 million infections, with a death toll exceeding 6.5 million as of September 20th, 2022 according to the World Health Organization COVID-19 dashboard (2).

1. The health emergency caused by the coronavirus pandemic dramatically changed clinical practice during the pandemic and beyond. The first wave impacted liver transplantation differently across the world, with particularly detrimental effects on the countries that sustained a severe hit by the virus (3). In 2020, The European Association for the Study of Liver disease (EASL), the European Liver and Intestine Transplant Association (ELITA) of the European Society of Organ Transplantation (ESOT), and the International Liver Transplantation Society (ILTS) task force demonstrated that the resilience of the entire transplant network enabled continued organ donation and transplantation, ultimately improving the lives of patients with end-stage liver disease (https://www.covid-19vaccinetracker.org/).

With dedicated financial support and worldwide commitment from the scientific community, on August 18th 2022, the WHO reported 276 vaccines in development, 109 in clinical trials, and 24 in use (4 with four prevalent types in the United States and Europe).

Currently, the data surrounding vaccination policies in liver transplant centers across the world are lacking. Consequently, EASL-ESOT-ELITA-ILTS task force convened and formulated a plan to investigate the variable approaches of liver transplant centers across the world in utilizing vaccines against SARS CoV-2 by means of an online survey. Herein, we report the results of this global survey, which may guide LT centers as they continue to operate and encounter the sequelae of the current pandemic and potentially optimize the future approach to vaccination.

## Materials/patients and methods

This is a cross-sectional survey aimed at exploring the approach to COVID-19 vaccines across adult liver transplant centers worldwide. A digital questionnaire composed of four sections assessing (i) vaccine policies, (ii) COVID-19 safety assessment, (iii) COVID-19 efficacy assessment, and (iv) center data was designed using the Google Surveys platform (https://surveys.google.com), including multiple choice and open question tools. Each section was composed of 6, 6, 2, and 3 questions, respectively (see Supplementary Table 7). The questionnaire was proposed by the authors and pretested among LT centers of the EASL-ESOT-ELITA-ILTS task force during a period of one month, correcting and clarifying the content. The final version of the digital survey was published online in October 2021 and remained available until February 2022. The global target population was obtained estimating the number of adult LT centers registered worldwide at the Global Database on Donation and Transplantation (GODT, http://www.transplant-observatory.org), and the corresponding physician staff were reached throughout the main international liver transplant societies (EASL, ESOT, ELITA, and ILTS) during the annual meeting and through their respective webpages. Furthermore, a periodic reminder promoting the survey was sent through the mailing list of the societies and was posted on Twitter and Facebook.

Results from the survey were integrated with the data from the Global Database on Donation and Transplantation to conceptualize information of COVID-19 vaccination in relation to global transplant activity.

A convenience sampling was applied, while sample size was not calculated given the exploratory purpose of the study. In order to prevent multiple participation, the e-mail and the name of the associated institution were required. Thus, double entries and unclear or incomplete data were resolved by contacting the participants. Responders that reported side effects of COVID-19 vaccination were re-contacted to clarify the type of side effect and the number of patients who suffered them.

The study was approved by the Medical Ethics Committee of Erasmus MC (MEC-2016-277). The study follows the CROSS guideline for survey studies (4).

## Statistical analysis

A survey analysis was performed, considering the hierarchical structure formed by three primary sampling units: (i) World Health Organization Regions, (ii) countries and (iii) LT centers, adjusted by the corresponding sampling weights. The sampling weights were obtained by considering 6 WHO Regions (https://www.who.int/countries), 86 countries with active LT programs, and 810 LT adult centers around the world (http://www.transplant-observatory.org). Stratification and finite population corrections were not used for the study. Data were presented by percentages and frequencies with the corresponding 95% confidence intervals. Distribution of continuous variables was assessed by the Shapiro–Wilk test. Comparisons were performed using multinomial and logistic regression according to their distribution. *P* values were expressed after Bonferroni correction in subgroup analyses.

A sensitivity analysis was performed by multiple imputation analysis of missing data that was considered missing completely at random. A multivariate chained model using predictive mean matching imputation was used to fit 100 imputations for each missing value. A *p*-value lower than 0.05 was considered statistically significant. Statistical analysis was performed using Stata 15 Software (StataCorp. 2017. Stata Statistical Software: Release 15. College Station, TX: StataCorp LLC).

## Results

### WHO regions responders

**Figure 1:**
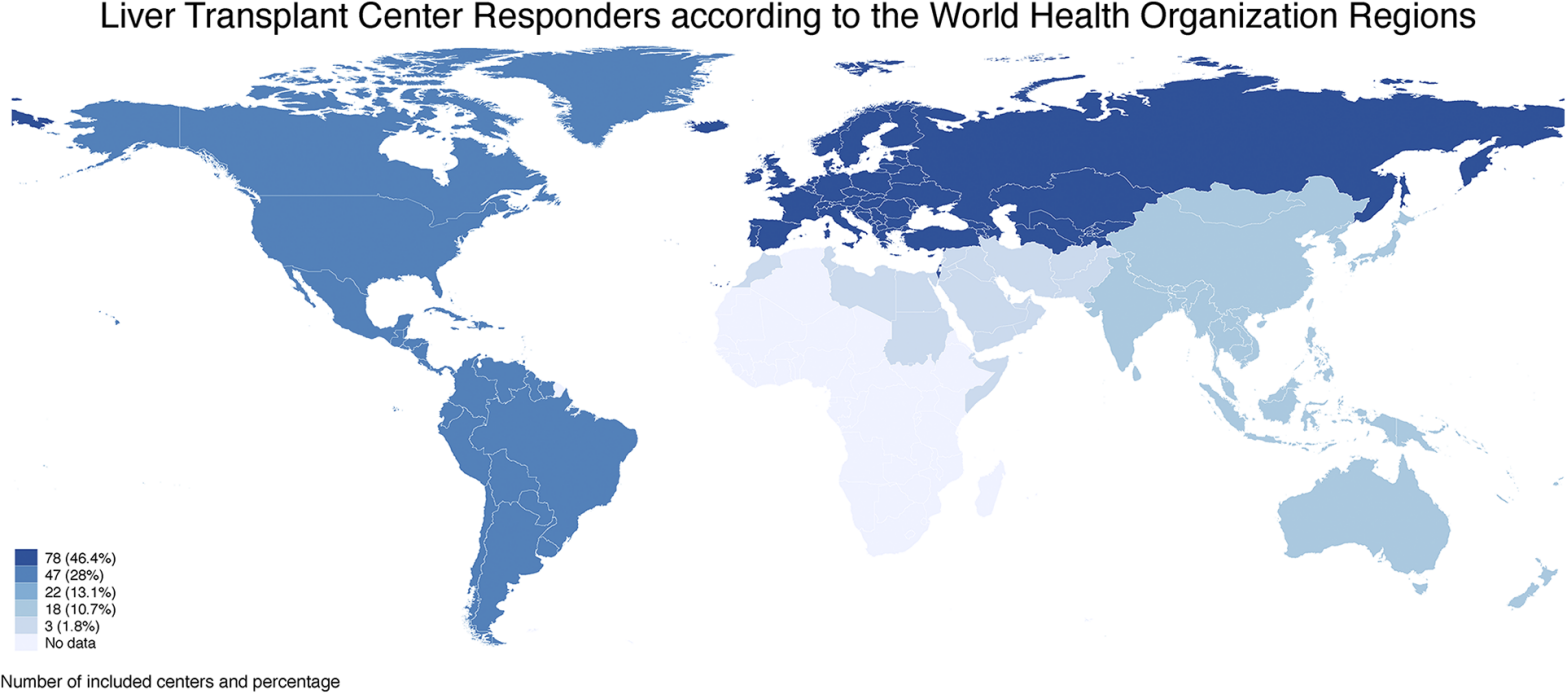
The number of liver transplant centers included in the survey are graphically represented with a blue scale according to the world health organization regions. European and American Regions had the highest number of included centers (78 and 47, respectively), followed by Western Pacific, South East Asian, and Eastern Mediterranean Regions with 22 (13.1%), 18 (10.7%) and 3 (1.8%) centers, respectively. No centers were included from African Regions.

According to the World Health Organization (WHO) Classification, the Regions that participated in the survey were European (46.4%, *n* = 78 Countries), American (28%, *n* = 47 Countries), Western Pacific (13.1%, *n* = 22 Countries), South East Asian (10.7%, *n* = 18 Countries), and Eastern Mediterranean (1.8%, *n* = 3 Countries) with a total of 168 corresponding centers, with no responders from African Regions (Figure 1).

### Country responders

Overall, 54.7% of the Countries in the World responded to this survey (47 out of 86 countries that have an LT program).

Of the 168 centers, United States of America (USA), Spain, India, and Italy were the most frequent centers that responded to the survey (17.9%, *n* = 31 centers, 13.3%, *n* = 23; 11%, *n* = 16; 9.25%, *n* = 16 centers, respectively) followed by Australia with 5 centers (2.9%); China, Germany, Japan, Russia, and Turkey with 4 centers (2.3%); Austria, Australia, Canada, Croatia, Mexico, and the United Kingdom with 3 centers (1.7%); Argentina, Brazil, Czech Republic, Egypt, France, Netherlands, Sweden, Taiwan, and Vietnam with 2 centers (1.2%); and Belgium, Chile, Colombia, Costa Rica, Finland, Georgia, Greece, Ireland, Malaysia, Nepal, New Zealand, Norway, Oman, Panama, Peru, Philippines, Romania, Thailand, and Trinidad and Tobago with 1 center (0.6%).

We estimated a total number of 810 adult active liver transplant (LT) centers across the included countries, excluding the Philippines and Taiwan where this information was lacking. Countries with 100% LT center participation were Australia, Austria, Czech Republic, Finland, Georgia, Greece, Ireland, New Zealand, Norway, Oman, Panama, Romania, Trinidad and Tobago, and Sweden, followed by Spain and Italy with 85.2% and 76.2%, respectively (Table 1).

**Table 1:** Liver transplant activity was summarized according to the World Health Organization Regions and Countries. Percentages of included centers per country were also described. The global activity per Regions and Countries were similar. However, the included centers represented approximately 21% of this population.

WHO Regions	LT in 2020/2021	Countries	LT in 2020/2021	Center included *n* (%)	Center per Country *n*/*N* (%)
AR	12,329/12,765	Argentina	316/438	2 (1.19%)	2/35 (5.71%)
		Brazil	2,075/1,944	2 (1.19%)	2/77 (2.6%)
		Canada	565/589	3 (1.79%)	3/7 (42.9%)
		Chile	127/125	1 (0.60%)	1/9 (11.11%)
		Colombia	199/229	1 (0.60%)	1/8 (12.5%)
		Costa Rica	15/20	1 (0.60%)	1/2 (50%)
		Mexico	72/135	3 (1.79%)	3/20 (15%)
		Panama	3/0	1 (0.60%)	1/1 (100%)
		Peru	17/20	1 (0.60%)	1/3 (33.3%)
		Trinidad & Tobago	0/0	1 (0.60%)	1/1 (100%)
		United State	8,901/9,236	31 (18.45%)	31/142 (21.8%)
ER	9,219/9,333	Austria	158/159	3 (1.79%)	3/3 (100%)
		Azerbaijan	45/NA	1 (0.60%)	1/3 (33.33%)
		Belgium	235/268	1 (0.60%)	1/6 (16.7%)
		Croatia	95/104	2 (1.19%)	2/3 (66.7%)
		Czech Republic	172/186	2 (1.19%)	2/2 (100%)
		Finland	75/75	1 (0.60%)	1/1 (100%)
		France	1,128/1,225	2 (1.19%)	2/26 (7.7%)
		Georgia	10/15	1 (0.60%)	1/1 (100%)
		Germany	826/834	4 (2.38%)	4/27 (14.8%)
		Greece	32/24	1 (0.60%)	1/1 (100%)
		Ireland	37/35	1 (0.60%)	1/1 (100%)
		Italy	1,202/1,396	16 (9.52%)	16/21 (76.2%)
		Netherlands	186/181	2 (1.19%)	2/3 (66.7%)
		Norway	88/98	1 (0.60%)	1/1 (100%)
		Portugal	193/202	2 (1.19%)	2/3 (66.7%)
		Romania	62/53	1 (0.60%)	1/1 (100%)
		Russia	559/524	4 (2.38%)	4/27 (14.8%)
		Spain	1,034/1,078	22 (13.10%)	23/27 (85.2%)
		Sweden	172/170	2 (1.19%)	2/2 (100%)
		Switzerland	135/151	2 (1.19%)	2/4 (50%)
		Turkey	1,320/1,528	4 (2.38%)	4/11 (36.36%)
		United Kingdom	823/821	3 (1.79%)	3/7 (42.9%)
WPR	8,160/7,778	Australia	277/254	5 (2.89%)	5/5 (100%)
		China	5,828/5,822	4 (2.38%)	4/114 (3.5%)
		Japan	380/421	4 (2.38%)	4/25 (16%)
		Malaysia	28/15	1 (0.60%)	1/2 (50%)
		New Zealand	55/53	1 (0.60%)	1/1 (100%)
		Philippines	0/0	1 (0.60%)	N/A
		South Korea	1,542/1,531	2 (1.19%)	2/40 (5%)
		Taiwan	543/508	2 (1.19%)	N/A
		Vietnam	NA/NA	2 (1.19%)	2/9 (22.2%)
SEAR	1,914/2,936	India	1,780/2,847	16 (9.52%)	16/95 (16.8%)
		Nepal	0/8	1 (0.60%)	1/3 (33.3%)
		Thailand	125/89	1 (0.60%)	1/9 (11.1%)
EMR	930/1,450	Egypt	395/515	2 (1.19%)	2/20 (10%)
		Oman	0/2	1 (0.60%)	1/1 (100%)
Total	32,552/34,262		31,830/33,928	168 (100%)	168/810^a^ (20.74%)

LT, liver transplant; WHO, World Health Organization; AR, American Regions; ER, European Regions; WPE, Western Pacific Regions; SEAR, South East Asia Regions; EMR, Easter Mediterranean Regions.

^a^ Estimated total given the lacking information on Taiwan and Philippines number of active centers and the possible discrepancy on real active Liver transplant Centers.

### Liver transplant activity in 2020 and 2021 across the world

The number of LT performed across the WHO Regions in 2020 and 2021 were 12,329 and 12,765 in American R. (AR), 930 and 1,450 in Eastern Mediterranean R. (EMR), 9,219 and 9,333 in European R. (ER), 1,914 and 2,936 in South East Asian R. (SEAR) and 8,160 and 7,778 in Western Pacific R. (WPR), respectively.

Overall, the median number of LT performed in 2020 and 2021 per Country was 172 (Q1-Q3, 45–565) and 181 (53–589), respectively, with an increase in 2021 (*p* = 0.043). Out of 47 Countries, 53.2% (*n* = 25) had a significantly increased number of LT, 36.2% (*n* = 17) a significantly lower transplant activity, and 6.4% (*n* = 3) similar activity (2 missing data, Table 1).

### Survey results

#### Vaccine policies

Most LT centers prioritized access to COVID-19 vaccines for LT patients (76%, 95%CI 41%–94.1%) and health care workers (86.2%; 67.3%–95%), while cohabitants, life partners of LT patients, and other categories were rarely included in these prioritization policies (26.2% and 13.5%, respectively). WPR, SEAR, AR, and ER were more likely to prioritize vaccination of LT patients than other regions.

Most LT centers had no specific recommendation about the type of COVID-19 vaccine administration (69.8%; 95%CI 43.6%–87.4%), while one-third restricted it just to mRNA vaccines (30.2%; 95%CI 12.6%–56.4%).

Booster (third) dose for mRNA Pfizer® vaccine was widely recommended in LT patients (80.5%; 95%CI 35.2%–96.9%). ER were more likely recommended mRNA vaccines for LT patients, while booster doses were largely implemented in American Regions compared with the other regions.

Approximately one-third of centers reported the presence of barriers for COVID-19 vaccine administration (31.8%, 95%CI 14.3%–56.4%), mainly because of patient concerns (89.4%; 48.8%–98.7%) and regional/national public health policies (35.7%; 95%CI 26%–46.7%). Regions with a higher presence of barriers were mainly EMR and WPR.

The main administering providers of COVID-19 vaccines were health-care and governmental authorities (61.9%; 95%CI 31.5%–85.1%) followed by transplant providers (25.3%; 95%CI 9.2%–52.9%) and primary care physicians (12.9%; 95%CI 3%–41.1%). WPR had a higher involvement of health care authorities in ordering COVID-19 vaccines.

Most centers routinely recommended other types of vaccines (e.g., flu) (89.4%; 95%CI 63.7%–97.6%) that were provided by transplant centers (56.8%; 95%CI 31.9%–78.6%) or by primary care clinics (43.2%; 95%CI 21.4%–68.1%). ER and AR were more likely to recommend other routine vaccinations, see Table 2 and Supplementary Table S1.

**Table 2:** Policies for COVID-19 vaccination showed that transplant patients and health care workers were prioritized in the vast majority of centers without a specific restriction for the type of vaccine and promoting a booster dose and other vaccines (e.g flu).

		Proportion	[95% Conf. interval]	
Categories of prioritized access	Transplant patients	0.768	0.410	0.941
	Health care workers	0.862	0.673	0.950
	Co-habitants/life	0.262	0.129	0.460
	Other categories	0.135	0.090	0.198
Type of vaccine recommended	No specific restrictions	0.698	0.436	0.874
	Only mRNA vaccines	0.302	0.126	0.564
	Booster (third) dose	0.805	0.352	0.969
Barriers for vaccination administration	Presence of any of the follow	0.318	0.143	0.564
	Patient's fear	0.894	0.488	0.987
	Regional or National Public health policy	0.357	0.260	0.467
	Age	0.146	0.035	0.444
	Logistics/Organization	0.106	0.019	0.421
	Funding	0.048	0.003	0.423
	Others	0.265	0.126	0.476
Facility ordering the vaccination	Health-care authorities	0.619	0.315	0.851
	Primary care physician	0.129	0.030	0.411
	Transplant provider	0.253	0.092	0.529
Other vaccines (flu, etc.) routinely recommended	Routinely recommended	0.894	0.637	0.976
	Provided by Primary Care Clinic	0.432	0.214	0.681
	Provided by Transplant Center	0.568	0.319	0.786

#### COVID-19 safety assessment

Approximately one-third of the responders implemented routine liver-related biochemical testing post COVID19 vaccine (29.6%; 95%CI 22.1%–38.4%), mainly at 2 and 4 weeks after its administration (38.2%; 95%CI 10.9%–75.8% and 37.6%; 95%CI 15.5–66.5%, respectively). Monitoring was reported mainly by outpatient clinics (62.7%; 95%CI 30.6%–86.5%) and via telemedicine (26.7%; 95%CI 8.7%–58.3%). Liver biochemical tests were mainly assessed by EMR and ER regions, while telemedicine was likely preferred by AR and SEAR.

A small proportion of centers modified the immunosuppression before COVID-19 vaccination (16.1%; 95%CI 5.9%–37.3%), with MF discontinuation or reduction being the most common approach (41.4%; 95%CI 10.4%–81.1% and 37.8%; 95%CI 7.6%–81.9%, respectively), followed by steroid reduction (29.4%; 95%CI 3.6%–82.1%). Preventive dose or type modification of IS were more frequent in SEAR and ER, as shown in Table 3 and Supplementary Table S2.

**Table 3:** Safety assessment after COVID-19 vaccination was scarcely implemented as well as preemptive immunosuppression modification, where mycophenolate adjustment was the most common approach.

		Proportion	[95%_Conf. interval]	
Liver function test (LFT) monitoring		0.296	0.221	0.384
Time of LFT monitoring	2 weeks	0.376	0.155	0.665
	1 month	0.382	0.109	0.758
	3 months	0.145	0.022	0.559
	Other	0.097	0.010	0.545
Via report of vaccine monitorization	Telemedicine	0.267	0.087	0.583
	Outpatient clinic	0.627	0.306	0.865
	Patient self-report	0.070	0.015	0.264
	Other	0.036	0.004	0.266
Type or dose of immunosuppression modification pre-vaccine		0.161	0.059	0.373
	CNI reduction	0.093	0.014	0.420
	MF reduction	0.378	0.076	0.819
	MF interruption	0.414	0.104	0.811
	Steroids reduction	0.294	0.036	0.821
	Steroids interruption	0.166	0.016	0.715
	Others	0.073	0.008	0.444

LFT, liver function test; CNI, calcineurin inhibitor; MF, mycophenolate.

Overall 35.7% (95%CI 20.9%–53.9%) of the centers reported any of the following significant adverse drug events (ADEs) after COVID-19 vaccine: 13.8% of centers (95%CI 6.6%–26.5%) reported ≥one occurrence of thrombosis or thromboembolic event, 13% (95%CI 2.8%–43.6%) acute rejection, 9.5% (95%CI 1.6%–40.7%) other significant liver related alterations different from isolated biochemical alteration, 4.3% (95%CI 0.9%–18.8%) allergic reaction and 14.6% (95%CI 8.2%–24.7%) other side effects. Moreover, 29.5% (95%CI 15.8%–47.7%) of centers reported LFT alterations. Out of 38 centers that reported side effects, only 14 specified the number of patients involved. Excluding liver-related biochemical alteration, the estimated rate of side effects per patient was about 1 case (95%CI 0%–3%) per 1,000 vaccinated patients. Significant side effects post-vaccination were more frequently reported in EMR, AR, and ER, see Table 4 and Supplementary Table S3.

**Table 4:** Side effects were reported in 36% of the centers, with thrombosis and thromboembolic events being the most relevant. The estimated rate of side effects excluding liver enzymes alteration was 1 case per 1000 of vaccinated patients. The vaccination efficacy was rarely evaluated, and 12% of the center reported ≥1 severe infection despite the vaccination.

Side effects post vaccination (questions 9–11)		Proportion	[95%_Conf. interval]	
Liver enzymes elevation		0.295	0.158	0.477
Thrombosis or thromboembolic event		0.138	0.066	0.265
Acute graft rejection		0.130	0.028	0.436
Liver related		0.095	0.016	0.407
Allergy related		0.043	0.009	0.188
Others significant adverse event^a^		0.146	0.082	0.247
Total side effects (except liver enzymes elevation)		0.357	0.209	0.539
Rate of significant side effects per patient^b^		0.001	0.000	0.003
Efficacy assessment (questions 12–13)				
Centers testing antibodies		0.252	0.065	0.621
Timing of test	2 weeks	0.062	0.010	0.310
	1 month	0.391	0.181	0.651
	3 months	0.275	0.141	0.468
	6 months	0.021	0.004	0.108
	Other	0.008	0.001	0.042
Infection post vaccine	Yes	0.221	0.072	0.508
	Severe	0.553	0.275	0.802

^a^ Myocardial infarction (*n* = 2); post-transplant lymphoproliferative disease (*n* = 2); cholangitis (*n* = 1); Guillain Barré (*n* = 1); leukopenia (*n* = 1); thrombocytopenia (*n* = 1) lymphadenitis (*n* = 1); retinal detachment (*n* = 1); menorrhagia/dysmenorrhea (*n* = 1); unknow (*n* = 1).

^b^ Estimation of significant side effects rate per patients based on 14 centers who specified the number of patients with side effects over 38 that reported any of them.

After adjusting for the WHO Regions, viral vector-based vaccines were significantly associated with a higher risk of thrombotic event (contrast 20.3; 95% CI 3.152, 37.48; *p* = 0.029). Combined mRNA plus viral vector formulation or other formulations were associated with lower risk of allergic side effects (−1.935, *p* = 0.072 and −21.082; *p* = 0.002, respectively). Furthermore, other complications different from liver biochemical alteration, thrombosis, acute rejection, and allergy were significantly higher in viral vector-based vaccines (23.553; *p* = 0.034), while combined formulation had a tendency for lower risk of these complications (−5.931, *p* = 0.059), see Table 5.

**Table 5:** Viral vector vaccines were significantly associated with a greater risk of thrombosis (*p* = 0.029) and other complications (*p* = 0.034), while acute rejection, allergy and liver related side effects were not associated with a specific type of vaccine.

	Side effects	Contrast	[95% Conf. interval]		*P* > *t*	Sig
mRNA (yes vs. mean)	All side effects	−1.685	−13.787	10.417	1.000	
Viral vector		8.183	−2.797	19.162	0.119	
Mix		−0.332	−4.518	3.855	1.000	
Other vaccine		−6.348	−20.824	8.128	0.400	
mRNA (yes vs. mean)	Thrombosis	4.319	−15.060	23.699	0.959	
Viral vector		20.316	3.151	37.482	0.029	**
Mix		−2.195	−12.939	8.549	1.000	
Other vaccine		−22.441	−44.024	−0.858	0.044	**
mRNA (yes vs. mean)	Acute rejection	−1.074	−36.103	33.956	1.000	
Viral vector		−9.876	−32.656	12.904	0.409	
Mix		6.237	−4.179	16.653	0.209	
Other vaccine		4.432	−27.971	36.834	1.000	
mRNA (yes vs. mean)	Allergy	14.868	−21.188	50.923	0.446	
Viral vector		8.149	−33.844	50.142	1.000	
Mix		−1.935	−4.113	0.244	0.072	*
Other vaccine		−21.082	−29.198	−12.966	0.002	***
mRNA (yes vs. mean)	Liver related	−0.322	−11.443	10.798	1.000	
Viral vector		−8.301	−23.078	6.476	0.242	
Mix		12.511	2.693	22.329	0.022	
Other vaccine		−4.185	−19.902	11.532	0.809	
mRNA (yes vs. mean)	Others	−8.618	−42.805	25.569	0.856	
Viral vector		23.553	2.633	44.472	0.034	**
Mix		−5.931	−12.170	0.308	0.059	*
Other vaccine		−9.195	−38.357	19.967	0.665	

* *p* < 0.1.

** *p* < 0.05.

*** *p* < 0.01.

#### Efficacy assessment

Approximately one-fourth of the centers tested for COVID-19 antibodies (25.2%; 95% CI 6.5%–62.1%), which was commonly carried out 1 and 3 months after its administration (39.1%; 95% CI 14.1%–46.8% and 27.5%; 95% CI 14.1%–46.8%, respectively). ER together with WPR were more likely to test for antibodies after COVID-19 vaccination.

Approximately half of the centers reported COVID-19 infections in LT patients despite vaccination (56.1%, 95% CI 25.7%–82.6%); among these, one-fourth had a severe infection (25.8%, 95% CI 7.3%–60.3%). COVID-19 infection post-vaccine was more frequent in EMR, followed by ER and AR, yet more severe infection was more likely reported in SEAR and AR regardless of the type of vaccine administered (see Table 4 and Supplementary Table S4).

#### Center data

The mean number of alive LT patients per center was 698.5 (504.3–892.6), yet the data were available in only 57.7% (*n* = 97) of responders.

Patients were mostly vaccinated 12 months after LT (73.8%; 95% CI 66.1%–81.4%). Only one-fourth of LT patients were vaccinated within 3 months post-transplant (25.1%; 95% CI 9.8%–40.4%).

COVID-19 vaccine formulation was mRNA-based (Pfizer®/Moderna®) in 49.6% (95% CI 17.5%–81.8%), viral vector (Astra-Zeneca®/Janssen®) in 30.8% (95% CI 1.5%–60.1%), and a mix of them in only 4.7% (95% CI 0.5%–8.9%) of cases. About one-sixth of the patients received a different formulation (15%; 95% CI −4.7% to 34.6%), mainly in SEA and ER (7/17 and 6/17 centers). Globally, AR, WPR, and ER were the regions with higher percentages of vaccinated patients; mRNA vaccine was largely preferred in ER and AR, while viral vector was more frequently used in EMR and SEAR (Table 6 and Supplementary Table S5).

**Table 6:** Data at the center level is summarized. The mean number of alive patients at the time of the survey was approximately 700. The percentage of patients fully vaccinated was around 87%. The most common type of vaccine was mRNA based in 50% of centers, followed by viral vector type in 31% of the centers.

		Proportion (*n*)	[95%_Conf. interval]	
LT patients alive (*n*)		698.459	504.334	892.584
Timing of vaccination administration	<3 months (%)	25.116	9.809	40.423
	3–6 months (%)	50.869	21.674	80.063
	6–12 months (%)	58.748	45.334	72.162
	>12 months (%)	72.682	66.977	78.388
Globally vaccinated (%)		86.570	81.550	91.590
Patients vaccinated (*n*)^a^		667.120	332.030	1,002.210
Type of vaccine administered (%)	mRNA	49.646	17.523	81.770
	Viral vector	30.811	1.515	60.107
	Viral vector and mRNA	4.695	0.502	8.888
	Others	14.968	−4.681	34.618

^a^ This estimation is based on the number of alive patients (available in 58.9%, *n* = 99) and the rate of fully vaccinated patients per center (available in 52.5%, *n* = 88).

### Sensitivity analysis

Missing data for LT patients alive, vaccinated for COVID-19, and suffering significant ADEs per each center were obtained by multiple imputation considering hierarchical LT activity (globally, WHO regions and centers) in 2020 and 2021, country, and the number of centers per country. A total of 144 centers had complete data (see Supplementary Table S6): the mean rate of LT alive patients per center was 697.08 (95%CI, 360.56–1,033), the mean rate of COVID-19 vaccinated patients was 87.1%(95% CI 71.75%–100%), the estimated number of patients vaccinated per center at the time of the survey was 620.48 (273.3–967.66), and the mean rate of patients with ADEs was 0.78 (95% CI 0–0.233) per 1,000 patients.

Given the estimated rate of ADEs, mRNA vaccines showed a higher safety profile compared with the remainder vaccines, with −3.998 (−6.466, −1.631; *p* = 0.013) cases per 1,000 vaccinated patients, yet vial vector vaccines showed a significant increase rate of ADEs once compared with the remainder (3.544, 95% CI 0.762; 6.326; *p* = 0.028).

## Discussion

Vaccination against COVID-19 is a major tool in the fight against the pandemic and has profoundly impacted its progression, with an estimated 14.4 million deaths worldwide prevented by vaccines during the first year of the vaccination program (from December 2020 to December 2021) (5). Liver transplant recipients are a high-risk group for COVID-19 infection, mainly due to their medical comorbidities, rather than to their immunosuppressed status. Scientific societies have recommended vaccination in LT recipients (6). However, access to vaccines and the related policies around the world have not been uniform.

This multi-society global survey is the largest study exploring current practices and policies regarding COVID-19 vaccination in LT centers across the world. A previous study (7) reported significant heterogeneity in COVID-19 vaccination mandate policies among centers in the US. In our study, most of the centers prioritized LT recipients and healthcare workers for COVID-19 vaccination and recommended a booster dose. This recommendation is in line with EASL and AASLD current recommendations (6, 8). However, cohabitants and life partners of LT recipients were rarely prioritized. A specific type of vaccine was not recommended in more than two thirds of programs (69.8%), and the main type of vaccine administered was an mRNA-based vaccine in approximately half of the studied centers. Importantly, concurrent routine vaccinations such as flu vaccination were recommended in most centers (89%). Of note, previous vaccination with flu has been described as a predictive factor for acceptance to receive vaccination against COVID-19 (9).

We also explored the different barriers to vaccination. The main reported barrier to vaccination was the patient's fear in 89% of cases. Vaccination hesitancy is another factor that is frequently encountered in the general population and has also been described in the transplant population (10). Counseling the patient about vaccine safety is crucial in order to increase the vaccination rate in transplant recipients. In more than one third of survey responses, public health policies were reported as a barrier to vaccination.

Interestingly, vaccination policies were slightly different in some regions; EM and WP Regions reported a higher presence of barriers and EMR had more difficulties in prioritizing COVID-19 vaccination in LT patients. Moreover, mRNA vaccines were more likely to be recommended in ER and a booster dose was more commonly implemented in AR. Finally, AR and ER were more prone to recommend other routine vaccinations.

Timing of COVID vaccination in LT recipients is not clearly defined, but it has been suggested that it should be performed after 3 months post-LT, when immunosuppression is lower. In our study, vaccination was mostly performed >12 months post-LT (73%). Only 25% of centers reported vaccination of patients in the first 3 months post-LT.

Efficacy assessment by COVID-19 antibodies post-vaccination at 1 and 3 months was limited, probably due to the paucity of data regarding the immune correlates of protection (CoP) after vaccination, the lack of a clear cut-off of neutralizing antibody titer against COVID-19, and the concern about the rapidly emerging variants with capacity to escape the specific antibody protection, especially in immunocompromised patients (11–13).

Preventive adjustment of immunosuppression regimen or dose before COVID-19 vaccination was uncommon across the assessed centers, occurring in just 16% of them. Among these, the most common approach was MF discontinuation or reduction of dose. This approach was probably driven by the known cytotoxic effect of MF on activated lymphocytes and the arising data showing absent or suboptimal antibody responses to COVID-19 vaccination in patients on MF (14, 15).

The most relevant side effects following COVID-19 vaccination were thrombosis or thromboembolic events, acute rejection, and significant liver dysfunction in 13.8%, 13%, and 9.5% of assessed centers. It is important to highlight that these rates are reflective of the number of centers that reported occurrence of ≥1 of these adverse events divided by the number of all participating centers in this survey and that these rates do not reflect the possibility of occurrence per patient. Interestingly, viral vector vaccines were associated with an increased risk of thrombotic events by 20.3 points (95% CI 3.152, 37.48; *p* = 0.029). Several reports showed an increased risk of splenic, porto-mesenteric, and hepatic thrombosis, cerebral arterial thromboembolism, and thrombotic microangiopathy (16–18) for adenoviral vector vaccines, contrary to observations for mRNA-based vaccines (19). Despite being unable to assess the incidence of thrombotic events, which based on the literature should be considered as a rare event (6), it provides evidence that the type of vaccine is highly relevant.

In order to better clarify the incidence and the type of side effects at the patient level, all centers that reported any of the aforementioned side effects were re-contacted. Unfortunately, the responders that specified the number of patients affected by side effects remained low (36.8%), precluding a clear estimation of its incidence. Thus, a sensitivity analysis using a complex imputation model replacing the missing data allowed for an estimate of a global ADEs incidence of 0.781 (95%CI 0; 0.233) per 1,000 vaccinated patients. This incidence is challenging to compare with what has been reported in literature as it reflects a pooled estimation of specific and unspecific side effects such as thrombosis, acute rejection, allergy, liver related, and others. Despite the fact that this model was unable to assess the specific incidence for each side effect, it globally confirmed that mRNA vaccine had a better safety profile compared with the other vaccine types (3 cases of ADEs less for every thousand vaccinated patients, *p* = 0.002).

The main limitations of this study are related to its nature, a cross sectional survey study. Indeed, we cannot rule out a selection bias with a relatively small proportion of centers worldwide that answered the questionnaire (20.7%). This challenges the extrapolation of the results, especially for WHO regions with low or no respondents (e.g., Eastern Mediterranean and African Regions). However, we estimated that the centers we interviewed account for approximately 45.6% of all liver transplant procedures performed in the included WHO regions during 2020 and 2021. Although the number of reported ADEs was double-checked directly with the corresponding center for case-by-case verification, the absence of ADE reports from the remainder centers relied on responder recall. The limited availability of patient level data is another limitation. Moreover, some of the questions had a low rate of response, perhaps because of the complexity in addressing the query. The study recognizes the challenges posed by the fast-changing landscape of vaccines and evolving guidelines compared to the 2021–2022 period. This study did not fully cover the latest COVID-19 vaccination recommendations for liver transplant patients, which now favor mRNA vaccines starting 3 months post-transplant (20). While viral vector vaccines are still advised in combination with mRNA vaccines, the focus has shifted towards mRNA vaccines. Nonetheless, the comprehensive nature of the analyses performed, including the sensitivity analysis addressing the missing data for the most relevant outcomes, provides valuable insights despite the acknowledged limitations.

In conclusion, this is the first multi-society survey assessing COVID-19 vaccination policies worldwide and it sheds light on the practices of LT centers in the new era of pandemic. Despite the obvious heterogeneity across WHO regions, this study was able to show that most centers prioritize COVID-19 vaccination in LT recipients and healthcare workers, recommending a booster dose and generally preferring mRNA-based vaccines, mainly administered 1 year after LT. There was no universal strategy to assess the efficacy of the vaccines or to adjust immunosuppression before COVID-19 vaccine administration, even though MF reduction or discontinuation was the most common approach. Finally, this study seems to confirm that adenoviral vaccine has an increased risk of thrombotic events, though it should be considered as a rare event.

## Data Availability

The raw data supporting the conclusions of this article will be made available by the authors, without undue reservation.
