# Supplementary material for: Worldwide variations in COVID-19 vaccination policies and practices in liver transplant settings: results of a multi-society global survey
Source: Front Transplant. 2024 Jan 19;2:1332616. doi: 10.3389/frtra.2023.1332616 (PMC11235330; doi:10.3389/frtra.2023.1332616)
Supplement: Supplementary file 1 [file Datasheet1.pdf]

Supplementary table 1: COVID19 Vaccine policies for Liver Transplant Patients (Questions 1-5)

		AR contr. vs mean; p	EMR contr. vs mean; p	ER contr. vs mean; p	SEAR contr. vs mean; p	WPR contr. vs mean; p
Prioritized access	Transplant patients	0.483; p<0.001	-1.331; p<0.001	0.418; p<0.001	0.969; p<0.001	1.686; p<0.001
	Health care workers	0.386; p<0.001	N/A	0.633; <0.001	-0.345; p<0.001	-1.066; p<0.001
	Co-habitants/life	-0.660; p<0.001	-1.861; p<0.001	0.493; p<0.001	0.440; p<0.001	0.554; p<0.001
	Other categories	N/A	N/A	N/A	N/A	N/A
Recommend vaccine	Only mRNA vaccines	-0.032; p<0.001	-2.361; p<0.001	0.634; p<0.001	N/A	-0.644; p<0.001
	Booster (third) dose	1.679; p<0.001	N/A	-0.206; p<0.001	-2.847; p<0.001	-0.930; p<0.001
Barriers	Presence of any of the follows	-0.774; p<0.001	3.814; p<0.001	0.377; p<0.001	-0.436; p<0.001	0.705; p<0.001
	Patient's Fear	-0.511; p<0.001	0.609; p<0.001	0.312; p<0.001	-0.490; p<0.001	0.036; p<0.001
	Regional or National Public Health Policy	0.460; p<0.001	N/A	-1.709; p<0.001	N/A	1.158; p<0.001
	Age	-0.839; p<0.001	1.389; p<0.001	0.073; p<0.001	0.290; p<0.001	N/A
	Logistics/Organization	0.350; p<0.001	2.109; p<0.001	-1.249; p<0.001	1.011; p<0.001	N/A
	Funding	1.700; p<0.001	N/A	-1.209; p<0.001	N/A	N/A
	Others	0.194; p<0.001	N/A	-0.478; p<0.001	N/A	0.285; p<0.001
Facility Ordering	Health-care authorities	-0.707; p<0.001	-0.856 p<0.001	-1.009 p<0.001	-1.305 p<0.001	3.053 p<0.001
	Primary care physician	-0.042 p<0.001	N/A	1.748 p<0.001	1.403 p<0.001	-2.532 p<0.001
	Transplant provider	1.075 p<0.001	1.505 p<0.001	0.286 p<0.001	0.992 p<0.001	-2.816 p<0.001
Other vaccines (flu, etc) routinely recommended	Routinely recommended	1.775 p<0.001	0.178 p<0.001	-0.003 p<0.001	-1.941 p<0.001	-1.316 p<0.001
	Provided by Primary Care Clinic vs Transplant Center	0.277 p=0.007	-17.112 p<0.001	0.848 p<0.001	-1.564 p<0.001	1.812 p<0.001

AR: American Regions; EMR: Ester Mediterranean Regions; ER: European Regions; SEAR: South East Asia Regions; WPR: Wester Pacific Regions

All results have been reported comparing each region with weighted grand mean equal to 0; p values have been expressed after Bonferroni correction, however all the results should be interpreted as trends.

Supplementary table 2: Assessment and Management in Liver Transplant Patients underwent COVID19 vaccination (questions 6-11)

		AR<br>contr. vs mean; p	EMR<br>contr. vs mean; p	ER<br>contr. vs mean; p	SEAR<br>contr. vs mean; p	WPR<br>contr. vs mean; p
Liver function test (LFT) or immunosuppression (IS) monitoring		-0.174; p<0.001	0.977; p<0.001	0.384; p<0.001	-0.299; p<0.001	-0.067; p<0.001
Time of LFT or IS monitoring	2 weeks vs 1 months	0.341; p=0.011	18.476; p<0.001	-1.290; p<0.001	-0.742; p=0.001	-2.757; p<0.001
Via report of vaccine monitorization	Telemedicine vs Outpatient Clinic	1.869; p<0.001	-17.132; p<0.001	0.570; p=0.002	1.907; p<0.001	-1.916; p<0.001
Dose of IS modification pre-vaccine		-0.397; p<0.001	N/A	0.720; p<0.001	1.224; p<0.001	-1.928; p<0.001
Type or dose of IS modification pre-vaccine	CNI reduction	0.732; p<0.001	N/A	-0.311; p<0.001	N/A; p<0.001	N/A
	MF reduction	-0.550; p<0.001	N/A	-0.773; p<0.001	1.229; p<0.001	N/A
	MF interruption	N/A	N/A	-0.490; p<0.001	0.599; p<0.001	N/A
	Steroids reduction	1.322; p<0.001	N/A	-0.562; p<0.001	N/A	N/A
	Steroids interruption	-0.259; p<0.001	N/A	0.134; p<0.001	N/A	N/A

AR: American Regions; EMR: Ester Mediterranean Regions; ER: European Regions; SEAR: South East Asia Regions; WPR: Wester Pacific Regions
LFT: Liver Function Test; CNI: Calcineurin Inhibitor; MF: Mycophenolate
All results have been reported comparing each region with weighted grand mean equal to 0; p values have been expressed after Bonferroni correction, however all the results should be interpreted as trends.

Supplementary table 3: Side Effects Post Vaccination per Center (Questions 8-11)

	AR contr. vs mean; p	EMR contr. vs mean; p	ER contr. vs mean; p	SEAR contr. vs mean; p	WPR contr. vs mean; p
Total Side Effect (except LFT elevation)	0.433; p<0.001	0.738; p<0.001	0.229; p<0.001	-0.234; p<0.001	-1.028; p<0.001
LFT elevation post-vaccination	0.606; p<0.001	-2.042; p<0.001	-0.264; p<0.001	-0.588; p<0.001	-0.098; p<0.001
Thrombosis or thromboembolic event	-0.449; p<0.001	-1.059; p<0.001	0.022; p<0.001	1.060; p<0.001	0.211; p<0.001
Acute graft rejection	1.327; p<0.001	N/A	-0.258; p<0.001	-0.432; p<0.001	-1.885; p<0.001
Allergy Related	-0.858; p<0.001	N/A	1.132; p<0.001	0.398; p<0.001	N/A
Significant Liver Related	0.579; p<0.001	N/A	-0.930; p<0.001	N/A	N/A
Others*	0.367; p<0.001	1.776; p<0.001	-0.804; p<0.001	-0.145; p<0.001	0.088; p<0.001
Estimate Rate of Significant ADE**	1.292; p<0.001	N/A	0.344; p<0.001	N/A	-2.740; p<0.001

AR: American Regions; EMR: Ester Mediterranean Regions; ER: European Regions; SEAR: South East Asia Regions; WPR: Wester Pacific Regions LFT: Liver Function Test;

** Over 38 centers that reported sides effects, only 36.8% (n=14) specified the number of patients having sides effects. The total number of patients affected by side effects was 23.*

*** Myocardial infarction (n=2); post-transplant lymphoproliferative disease (n=2); Cholangitis (n=1); Guillain Barré (n=1); Leucopenia (n=1); Thrombocytopenia (n=1) Lymphadenitis (n=1); Retinal detachment (n=1); menorrhagia/dysmenorrhea (n=1); unknow (n=1).*

All results have been reported comparing each region with weighted grand mean equal to 0; p values have been expressed after Bonferroni correction, however all the results should be interpreted as trends.

Supplementary table 4: Efficacy Assessment (questions 12-13)

		AR contr. vs mean; p	EMR contr. vs mean; p	ER contr. vs mean; p	SEAR contr. vs mean; p	WPR contr. vs mean; p
Centers Testing Antibodies		-0.863; p<0.001	-1.661; p<0.001	1.579; p<0.001	-0.682; p<0.001	0.463; p<0.001
Timing of antibody testing	1 month	-0.074; p<0.001	N/A	0.500; p<0.001	0.506; p<0.001	-1.209; p<0.001
	3 months	1.148; p<0.001	N/A	0.026; p<0.001	N/A	-0.760; p<0.001
Infection post vaccine		0.484; p<0.001	2.793; p<0.001	0.520; p<0.001	-0.400; p<0.001	-2.208; p<0.001
	Severe infection	0.826; p<0.001	N/A	-0.553; p<0.001	1.225; p<0.001	N/A

AR: American Regions; EMR: Ester Mediterranean Regions; ER: European Regions; SEAR: South East Asia Regions; WPR: Wester Pacific Regions LFT: Liver Function Test; All results have been reported comparing each region with weighted grand mean equal to 0; p values have been expressed after Bonferroni correction, however all the results should be interpreted as trends.

Supplementary table 5: Center data (questions 14-15)

		**AR**	**EMR**	**ER**	**SEAR**	**WPR**
		**contr. vs mean; p**	**contr. vs mean; p**	**contr. vs mean; p**	**contr. vs mean; p**	**contr. vs mean; p**
LT patients alive (n)*		-57.684	-437.959	-3.146	-116.313	252.456
Timing of vaccination administration	<3 months	9.031	-19.402	-8.253	9.538	-13.089
	3-6 months	21.809	-35.414	-0.201	-18.710	-19.835
	6-12 months	10.321	-38.748	-4.219	-5.391	-4.493
	>12 months	3.188	-18.137	-0.215	-9.323	1.933
Globally Vaccinated (%)		3.700	-19.229	1.083	-11.851	3.239
Patients Vaccinated (n)*		-88.158	-432.388	-83.250	64.957	297.549
Type of vaccine administered	mRNA	7.874	-40.555	27.166	-43.288	-9.155
	Viral Vector	4.369	41.916	-27.401	37.249	-8.201
	Viral vector and mRNA	2.472	-4.695	-2.392	-4.081	2.282
	Others	-14.506	3.213	2.505	9.999	14.954

AR: American Regions; EMR: Ester Mediterranean Regions; ER: European Regions; SEAR: South East Asia Regions; WPR: Wester Pacific Regions LFT: Liver Function Test;
*All results have been reported comparing each region with weighted grand mean equal to 0; p values have been expressed after Bonferroni correction, however all the results should be interpreted as trends. * This estimation is based on the number of alive patients (available in 58.9%, n=99) and the rate of fully vaccinated patients per center.*

Supplementary table 6: missing variables addressed by multiple imputations

Variables	Complete	Incomplete	Imputed	Total
Number of liver transplant patients	99	69	45	168
Number of vaccinated patients	88	80	52	168
Number of patients presenting side effects after vaccination	122	46	22	168
