# Supplementary material for: Worldwide variations in COVID-19 vaccination policies and practices in liver transplant settings: results of a multi-society global survey
Source: Front Transplant. 2024 Jan 19;2:1332616. doi: 10.3389/frtra.2023.1332616 (PMC11235330; doi:10.3389/frtra.2023.1332616)
Supplement: Supplementary file 2 [file Datasheet2.pdf]

Country	Center	Collaborators
Argentina	Hospital Italiano de Buenos Aires	E. Mauro, MD - L. Barcan, MD
Argentina	Consultorio	
Brazil	Unicamp	R. Stucchi, MD - I. Boin, MD
Brazil	Hospital do Rocio	F. Silveira, MD
Canada	Centre Hospitalier de l'Université de Montréal	I. Ruiz, MD - G. Huard, MD
Canada	Toronto General Hospital	
Canada	Queen Elizabeth II Health Sciences Centre, Halifax	K. M. Peltekian, MD, FRCPC
Chile	Pontificia Universidad Catolica de Chile	J. P. Arab, MD - L. A. Díaz, MD
Colombia	Centro Médico Imbanaco de Cali	A. Vanin, MD
Costa Rica	Hospital Mexico	
Mexico	Hospital General de Mexico	V. Visag-Castillo, MD - V. Páez Zayas, MD
Mexico	National Institute of Health Sciences and Nutrition Salvador Zubirán	N. C. Flores García, MD
Mexico	Hospital CMNO IMSS	
Panama	Caja de Seguro Social - Complejo Hospitalario Metropolitano	J. Lombardo MD - M. Spence MD
Peru	Transplant Department. Guillermo Almenara National Hospital	P. M. Padilla- Machaca MD, PhD, FAASLD - B. Cárdenas MD
Trinidad and Tobago	EWMSC/NOTU	
USA	TNVU - Vanderbilt University Medical Center - Nashville	
USA	MNUM - University of Minnesota Medical Center, Fairview	
USA	Mont Sinai New York	
USA	Intermountain Healthcare - Intermountain Medical Center	D. Alonso, MD - R. Gilroy, MD
USA	Ochsner Medical Center	
USA	Thomas Jefferson University Hospital	
USA	Northwestern University	
USA	Keck Medicine of USC	J. A. Kahn, MD
USA	Intermountain Medical Center	A. G. Contreras, MD
USA	Emory	
USA	University of Nebraska Medical Center - Omaha, NE	
USA	University of Rochester	
USA	UPMC Hospital of Pittsburgh	
USA	UTMC - University of Utah Medical Center, Salt Lake City	
USA	Aurora St Luke’s Medical Center, Milwaukee	
USA	Barnes Hospital	
USA	University of Chicago Medicine	
USA	Cleveland Clinic	
USA	University of Colorado	M. Kriss MD - L. Schoch, MSN, RN, CCTC
USA	OHOU - Ohio State University Medical Center	

USA	University of Rochester/ Strong Memorial Hospital	
USA	TNMH - Methodist University Hospital - Memphis	
USA	University of Minnesota	V. A. Kirchner, MD
USA	Mayo Minnesota	K. D. Watt, MD
USA	Mayo Florida	
USA	Medstar-Georgetown University Hospital	
USA	Montefiore Medical Center	
USA	Louis and Houston Methodist Hospital, HMH	
USA	University of Arkansas for Medical Sciences	E. Giorgakis, MD - L. Burdine MD
USA	University of Maryland	
USA	University of California, Los Angeles (UCLA)	
Egypt	National Liver Institute	
Egypt	Ain Shams University Center for Organ Transplantation	M. El Meteini,MD - I. Montasser, MD Professor
Oman	Royal Hospital	S. Al Zaabi, MD
Austria	Innsbruck	
Austria	Vienna	G. Berlakovich MD, Professor - G. Gyoeri, MD
Austria	Akh	
Azerbaijan	Azerbaijan Medical Universty	N. Bayramov, Prof. MD
Belgium	Not Avilable	
Croatia	University Hospital Center Zagreb	A. Mrzljak, MD - M. Premuzic, MD
Croatia	UHC Zagreb Liver Transplant Unit	M. Kovačić, MD - J. Vuković, MD
Czech Republic	Prague Transplant Program	J. Havlin, MD - T. Kotowski, MD
Czech Republic	University Hospital Hradec Kralove	
Finland	Helsinki	
France	Paul Brousse	I. Kounis, MD - B. Roche, MD
France	Montpellier	L. Meunier, MD
Georgia	Batumi Referral Hospital-liver transplant center	S. Beridze, MD, PhD - K. Kashibadze, MD, PhD
Germany	Leipzig	T. Berg, MD, Professor
Germany	UKSH, Campus Kiel	
Germany	Tübingen	
Germany	Hannover	
Greece	General Hospital of Athens “Laiko”	N. Machairas, MD - G. C. Sotiropoulos, MD
Ireland	Dublin	
Italy	Federico II University Hospital	M. Guarino, MD - V. Cossiga, MD
Italy	Papa Giovanni XXIII, Bergamo	S. Fagiuoli, MD - L. Pasulo, MD
Italy	Palermo ISMETT	
Italy	Azienda Policlinico, University of Catania	

Italy	University Hospital of Padova	
Italy	Tor Vergata University Hospital	R. Angelico, MD - T. M. Manzia, MD
Italy	Niguarda Hospital	L. S. Belli, MD - G. Perricone, MD - L. De Carlis, MD - R. De Carlis, MD
Italy	Molinette Hospital	
Italy	Naples University Hospital	
Italy	University Hospital Policlinico of Bari	A. Di Leo, MD - L. G. Lupo, MD
Italy	La Sapienza Hospital	Q. Lai, MD - M. Rossi, MD
Italy	Policlinico Maggiore of Milan	M. F. Donato, MD - C. Dibenedetto, MD
Italy	Policlinico Gemelli Hospital	M. M. Pascale, MD - G. Bianco, MD - S. Agnes, MD
Italy	IRCCS Azienda Ospedaliero-Universitaria Bologna	M. Cescon, MD - M. Ravaioli, MD
Netherlands	UMCG (Groningen)	V. E. De Meijer, MD
Netherlands	Rotterdam - Erasmus MC	
Norway	Oslo University Hospital	
Portugal	Centro Hospitalar Universitário do Porto	
Portugal	Unidade de transplantação hepática de adultos, Coimbra	S. M. N. Calretas, MD
Romania	Clinical Institute for Transplantation	
Russia	Almazov National Médical Research Centre	M. Simonenko, MD
Russia	Moscow Clinical Scientific Center	R. Alikhanov, MD - E. Sbikina, MD
Russia	Moscow Regional Scientific Research Clinical Institute n.a. M.F. Vladimirsky	K. Kokina, MD - Y. Moysyuk, MD
Russia	Burnasyan Federal Medical Biophysical Center of the Federal Medical Biological Agency	S. E. Voskanyan, MD - A. I. Sushkov, MD
Spain	Santiago, Hsopital clinico Universitario de Santiago	
Spain	Murcia, Clinic and University Virgen de la Arrixaca Hospital	
Spain	Valladolid, Hospital Universitario Rio Hortega	F. García Pajares, MD - C. Alonso Martín, MD
Spain	Navarra, Clinica Universidad de Navarra	J. I. Herrero, MD - M. Iñarrairaegui, MD
Spain	Cataluña, Vall Hebron	I. Campos-Varela, MD - L. Castells, MD
Spain	Tenerife, Hospital Universitario Ntra. Sra. de Candelaria	R. Suárez Darias, MD
Spain	Valencia, Hospital La Fe	C. Vinaixa, MD - M. Berenguer, MD
Spain	Paises Bascos, Cruces University Hospital	J. Bustamante Schneider, MD - P. Salvador Bengoechea, MD
Spain	Madrid, Hospital Gregorio Marañón	A. Caballero Marcos, MD - J. A. López-Baena, MD
Spain	Asturias, H. Universitario Central de Asturias	M. L. Gonzalez-Dieguez, MD - V. Cadahia, MD
Spain	Santander, Hospital Universitario Marqués de Valdecilla	
Spain	Madrid, Puerta de Hierro	V. Cuervas-Mons, MD - A. Arias, MD
Spain	Alicante, HGU Alicante	
Spain	Badajoz, Hospital Universitario de Badajoz	G. Blanco Fernández, MD - I. Narváez Rodríguez, MD
Spain	Madrid, Hospital Ramón y Cajal	R. Martín Mateos, MD - J. Graus Morales, MD
Spain	Zaragoza, Hospital Clínico Universitario Lozano Blesa	S. Lorente Pérez, MD - T. Serrano Aulló, MD
Spain	Madrid, Hospital Universitario La Paz	E. Frauca, MD

Spain	Sevilla, Hosp Univ Virgen del Rocío	G. Bravo Miguel Angel, MD - A. Martinez Jose María, MD
Spain	Cordoba, Hospital Reina Sofía	M. Rodríguez-Perálvarez, MD - M. De la Mata, MD
Spain	Cataluña, Hospital Universitari Bellvitge	L. Llado, MD - A. Cachero, MD
Spain	Cantabria, University Hospital Marqués de Valdecilla	
Spain	A Coruña, Hospital A Coruña	F. Suárez, MD - M. A. Vazquez, MD
Sweden	Karolinska	G. Soderdahl, MD
Sweden	Gothenburg	M. Oltean, MD - A. Schult, MD
Switzerland	Centre Hospitalier Universitaire Vaudois	
Switzerland	Inselspital	
Turkey	Inonu University	
Turkey	Umraniye Health Sciences University	G. Adali, MD - N. M. Bilgic, MD
Turkey	Ankara City Hospital	D. Turan, MD
Turkey	Medipol University Hospital	M. Dayangac, MD - S. Kilic, MD
UK	King's	
UK	Royal Free	
UK	Birmingham	
India	Secunderabad	
India	Amrita Institute of Medical Sciences and Research Centre	S. Sudhindran, MD - K. Nair, MD
India	Indraprastha Apollo Hospital	A. Malik, MD
India	Metro Multi-speciality Hospital	
India	Apollo Gujarat	
India	IKDRC-ITS, Ahmedabad	
India	Narayana Hospital	
India	AIG Hospitals	
India	Gleneagles Global Hospital	
India	Max Superspeciality Hospital, Saket	
India	Ruby hall clinic	
India	Medanta The Medicity	P. Bhangui, MD- A. S. Soin, MD
India	Apollo Tamil Nadu	
India	Jupiter hospital, Pune	P. Hanchanal, MD
India	SMS Medical College and Hospital	
India	Dr. Rela Institute & Medical Centre	A. Rammohan, MD - M. Rela, MD
Nepal	Chitwan Medical Collage	S. K. Yadav, MD - S. Poudyal, MD
Thailand	Chiang Mai University	S. Junrungsee, MD
Australia	Westmead Hospital	M. Stormon, MD
Australia	Flinders Medical Centre	
Australia	Princess Alexandra Hospital	C. Tallis, MD

Australia	Austin Health	
Australia	Royal Prince Alfred Hospital	S. Strasser, A/Professor
China	The First Affiliated Hospital of Nanjing Medical University	
China	Beijing Friendship Hospital	
China	First Affiliated Hospital, Nanjing medical University	
China	Beijing Tsinghua Changgung Hospital	J. Liu, MD
Japan	Nagoya University Hospital	Y. Ogura, MD
Japan	Tokyo Women's Medical University	H. Egawa, MD - S. Yamashina, MD
Japan	Center for Organ Transplantation	M. Kasahara, MD - S. Sakamoto, MD - A. Fukuda, MD
Japan	Kanazawa University	
Malaysia	University Malaya Medical Centre	Y. B. Koon, MD
New Zealand	New Zealand Liver Transplant Unit	
Philippines	The Medical City	
South Korea	Samsung Medical Center	J. M. Kim, MD - J. W. Joh, MD
South Korea	Seoul National University College of Medicine	
Taiwan	National Taiwan University Hospital	Cheng-Maw Ho, MD - Rey-Heng Hu, MD
Taiwan	Kaohsiung Chang Gung Memorial	
Vietnam	Organ Transplantation Center Viet Duc Univeristy Hospital	N. Q. Nghia, MD
Vietnam	108 Military Central Hospital - Departement of Hepatobiliary and Pancreatic Surgery	T. L. Van, MD
