# Supplementary material for: Worldwide variations in COVID-19 vaccination policies and practices in liver transplant settings: results of a multi-society global survey
Source: Front Transplant. 2024 Jan 19;2:1332616. doi: 10.3389/frtra.2023.1332616 (PMC11235330; doi:10.3389/frtra.2023.1332616)
Supplement: Supplementary file 3 [file Datasheet3.docx]

# LIVER TRANSPLANTATION – COVID VACCINATION SURVEY

**IDENTIFICATION:** Country/Region and Center

**VACCINATION POLICIES**

- Regarding national/regional regulation in access to vaccination

1. Are the following groups prioritised?
   - transplant patients (Y/N)
   - co-habitants/life partners of transplant patients (Y/N)
   - health care workers (taking care of transplanted patients) (Y/N)
2. Type of vaccine recommended for transplant recipients by your regional / national health authorities
   - All types (no specific restrictions)
   - Only mRNA vaccines
3. If vaccinated with mRNA Pfizer vaccine, is a third dose being administered to transplanted patients? (Y/N)
4. Are you aware of any barriers to vaccination in LT patients? (Y/N). If so, which type of barrier?
   - patient’s fear
   - funding
   - logistics/organisation
   - regional/national public health policy
   - age
   - gender

4) Who is ordering the vaccination of LT recipients

- Health-care authorities / governmental order
- Primary care physician
- Transplant provider

5) Are other vaccines (flu, etc ) routinely recommended for transplant recipients at your center (Y/N) and ordering clinic (transplant center vs primary care clinic)

**SAFETY ASSESSMENT:**

6) Regarding center monitoring policies, has your center implemented any specific monitoring policy to test for liver function tests (LFT) and/or immunosuppression (IS) levels post-vaccination?... Y/N

If so: at what time interval post-vaccination? (2 weeks, 1 month, 3 months?)

- via “telemedicine” vs oupatient clinic vs patient self-report

7) Are you modifying type / dose of IS before vaccination? If so, how?

- calcineurin inhibitor (CNI) dose reduction? …. Y/N

- CNI interruption? ….Y/N

- mycophenolate mophetil (MMF) dose reduction? ….Y/N

- MMF interruption? dose reduction? ….Y/N

- switch from MMF to mTOR inhibitors? ….Y/N

- steroids dose reduction? ….Y/N

- steroids interruption? …. Y/N

- others? …Y/N

Regarding observed adverse events:

8) Have you observed any case of LFT elevation post-vaccination?….Y/N

9) Have you observed any thrombosis or thromboembolic event postvaccination/N

10) Have you observed any case of acute graft rejection postvaccination/N

11) Have you observed any other (significant) adverse event post-vaccination?…. Y/N

- Allergy related….Y/N
- Liver related….Y/N
- Others….Y/N

**EFFICACY ASSESSMENT:**

12) Are you testing for antibodies post-vaccination? ….Y/N

- If so, how frequently post vaccination? (2 weeks, 1 month, 3 months?)

13) Have you had any patient diagnosed with COVID post-vaccination….Y/N

If so, was COVID-19 severe? ….Y/N

**CENTER DATA:**

14) Number of LT recipients alive in your center

15) Number (%) of LT recipients vaccinated within

- first 3 months post-LT
- 3-6 months post-LT
- 6-12 months post-LT
- >12 months post-LT

16) Type of COVID vaccine administered

- mRNA (Pfizer/ Moderna) .….% patients

- Viral vector (Astra-Zeneca/ J&J/Janssen) ….% patients
